# Identification of new Brazilian allergens from manioc (*Manihot esculenta*)

**DOI:** 10.1186/2045-7022-1-S1-O7

**Published:** 2011-08-12

**Authors:** Keity Santos Souza, Clovis Galvao, Resende Ferreira, Maria Virginia, Carlo Martins, Eva Vevjar, Gabriele Gadermaier, Fatima Ferreira, Jorge Kalil, Fabio Fernandes Morato-Castro

**Affiliations:** 1 Medical School of Sao Paulo University (FMUSP), Discipline of Allergy and Immunology - INCOR, Sao Paulo, Brazil; 2 Clinics Hospital of FMUSP, Service of Allergy and Immunology, Sao Paulo, Brazil; 3 University of Salzburg, Christian Doppler Laboratory for Allergy Diagnosis and Therapy, Department of Molecular Biology, Salzburg, Austria

## 

Brazil is a very rich country in terms of biodiversity offering broad spectra of possibilities to study new allergens, particularly from foods. In poor and developing countries manioc is a major source of carbohydrates in the diet of millions of people. In Brazil is the ninth cultivated food and the country takes the second place in the world as manioc cultivator. Allergic reactions to manioc were previously reported, but the allergen molecules involved and the cross-reactivity with other plant food sources or latex remain unknown. The aim of present work is to identify new manioc allergens, clone and express them. Based on clinical history, nine patients allergic to manioc were submitted to prick-to-prick test and basophil activation tests and sera were used for immunoblot analysis and ISAC. All patients demonstrated positive IgE reactivity to manioc proteins of approximately 30 and 40 kDa in 1D gel electrophoresis. Using 2D analysis, 6 immunoreactive spots were identified as allergenic-related protein Pt2L4, GADPH and fructose-bisphosphate-aldolase. Three out of five tested sera showed IgE reactivity to Hev b 5 in ISAC. The rich glutamic acid protein, Pt2L4 shows around 40% sequence identity with Hev b5 and 2 of 5 linear IgE epitopes identified for Hev b 5 are partially conserved in Pt2L4. Therefore, the mature sequence was amplified from manioc cDNA using specific primers and cloned into a pET-based expression vector. Clones from two different cultivars were checked and they are all the same sequence but deviate 3 amino acids from the three previously published sequences. Recombinant allergenic-related protein was expressed in E. coli BL21 Star and purified by ion exchange and size exclusion chromatography. Protein will be characterized regarding its physicochemical properties using aminoacid analysis, mass spectrometry, circular dichroism and simulated duodenal digestion. The IgE-binding activity of the recombinant molecule is assessed by IgE immunoblot and ELISA. In addition, IgE cross-reactivity with purified natural Hev b 5 is evaluated using sera of manioc and latex allergic patients.

## Support

FAPESP, INCT-iii

